# A reinvestigation of cognitive styles in sticklebacks: decision success varies with behavioral type

**DOI:** 10.1093/beheco/arae097

**Published:** 2024-11-25

**Authors:** Nick A R Jones, Kirstin Gaffney, Giacomo Gardella, Annie Rowe, Helen C Spence-Jones, Amelia Munson, Tom M Houslay, Mike M Webster

**Affiliations:** Department of Animal Physiology, University of Bayreuth, Universitätsstraße 30, 95447, Bayreuth, Germany; Centre for Social Learning and Cognitive Evolution, School of Biology, University of St Andrews, St Andrews, Fife, KY16 9TH, United Kingdom; Dove Marine Laboratory, School of Natural and Environmental Sciences, Newcastle University, Cullercoats, North Shields, NE30 4PZ, Newcastle Upon Tyne, United Kingdom; Centre for Social Learning and Cognitive Evolution, School of Biology, University of St Andrews, St Andrews, Fife, KY16 9TH, United Kingdom; Centre for Social Learning and Cognitive Evolution, School of Biology, University of St Andrews, St Andrews, Fife, KY16 9TH, United Kingdom; School of Liberal arts and Natural Sciences, University of Birmingham, Edgbaston, B15 2TT, Birmingham, United Kingdom; School of Biodiversity, One Health and Veterinary Medicine, University of Glasgow, Graham Kerr Building, Glasgow G12 8QQ, United Kingdom; Department of Wildlife, Fish and Environmental Studies, Swedish University of Agricultural Sciences, Skogsmarksgränd, SE-907 36, Umeå, Sweden; Independent Researcher, Manchester, United Kingdom; Centre for Social Learning and Cognitive Evolution, School of Biology, University of St Andrews, St Andrews, Fife, KY16 9TH, United Kingdom

**Keywords:** animal personality, behavioral trait, cognition, decision-making, methods, speed-accuracy trade-offs

## Abstract

The “cognitive styles” hypothesis suggests that individual differences in behavior are associated with variation in cognitive performance via underlying speed-accuracy trade-offs. While this is supported, in part, by a growing body of evidence, some studies did not find the expected relationships between behavioral type and cognitive performance. In some cases, this may reflect methodological limitations rather than the absence of a true relationship. The physical design of the testing arena and the number of choices offered in an assay can hinder our ability to detect inter-individual differences in cognitive performance. Here, we re-investigated the cognitive styles hypothesis in threespine stickleback (*Gasterosteus aculeatus*), adapting the maze design of a previous study which found no cost to decision success by faster (bolder) individuals. We used a similar design but increased the size of the maze and incorporated an additional choice in the form of a third maze arm. We found, in accordance with cognitive style expectations, that individuals who were consistently slower to emerge from the start chamber made fewer errors than fish that emerged faster. Activity in an open field test, however, did not show evidence of a relationship with decision success, possibly due to the low number of repeated observations per fish in this separate assay. Our results provide further empirical support for the cognitive styles hypothesis and highlight important methodological aspects to consider in studies of inter-individual differences in cognition.

## Teaser

Inter-individual variation in cognitive ability is hypothesized to be associated with behavioral traits through trade-offs between speed and performance. Previous research testing a common model species—the stickleback—did not find evidence to support the expected relationship between decision success and behavioral type. Using a more challenging test, with an additional choice and larger maze, we found that individuals that emerge faster (i.e. “bolder”) were also more error-prone, as predicted by the cognitive style hypothesis.

## Introduction

Inter-individual variation in behavior, including cognitive performance, has important ecological and evolutionary consequences. Individuals within a population often show consistent or repeatable differences in behavior relative to other individuals, such that individuals can exhibit a measurable “behavioral type” (Laskowski et al. 2022), (a.k.a. “personality”; Dall et al. 2012). These individual differences in behavior occur across a continuum for a range of behaviors, including exploration, level of activity, and social tendency and can profoundly shape behavioral responses across multiple ecological contexts in many species (Bell et al. 2009; Webster and Ward 2011; Dall et al. 2012; Laskowski and Bell 2014; Jessop et al. 2024). Such behavioral differences also have important consequences in cognition. Here, individual differences in behavior are perceived to act as non-cognitive influences on an individual, such that cognitive performance can vary across individuals with different behavioral types (Carere and Locurto 2011; Rowe and Healy 2014; Boogert et al. 2018; Lucon-Xiccato et al. 2019). However, while behavioral differences and cognitive performance are linked, the magnitude and shape of this relationship are not always clear across studies (Medina-García et al. 2017; Zidar et al. 2017; Dougherty and Guillette 2018).

The correlation between variation in cognitive performance and individual differences in behavior is frequently related to speed–accuracy trade-offs. Specifically, it is hypothesized that the behavioral type of an individual is linked to its cognitive performance via underlying trade-offs between speed and accuracy such that individuals exhibit measurable “cognitive styles” (Chittka et al. 2009; Carere and Locurto 2011; Sih and Del Giudice 2012; Heitz 2014). There is empirical support for the existence of a trade-off between speed and accuracy on cognitive tasks, most notably from a study that explored decision-making in bumblebees (*Bombus terrestris)* in a task where they had to select between rewarding and unrewarding artificial flowers (Chittka et al. 2003). Individual bumblebees that selected flowers more quickly were more error-prone across experiments, while slower bees were consistently more accurate. Similar findings have been found in studies across diverse species (Birds: Ducatez et al. 2015; Moiron et al. 2016; Mammals: Mazza et al. 2018; Fish: Latty and Beekman 2011; Gibelli et al. 2019; Lucon-Xiccato et al. 2019; and slime molds ).

The cognitive styles hypothesis provides a framework for exploring the relationship between individual differences in behavior and cognitive performance (Sih and Del Giudice 2012). This hypothesis can be viewed as comprising 2 main predictions. First, in associative learning: individuals that, on average, exhibit lower levels of neophobia, greater activity levels, and emerge from shelter more quickly are predicted to be faster learners on simple association tasks. These individuals with “fast-style” behavioral types are expected to encounter cues more quickly and develop associations more rapidly. Second, in more challenging cognitive tasks (such as when choosing and discriminating between multiple cues with differing rewards): faster individuals are predicted to be more prone to errors. Conversely, “slow-style” individuals are expected to perform better when discriminating between cues as they exhibit greater inhibition and attention and take more time before making a decision. However, empirical support for the second prediction and the expected relationships between the behavioral type of an individual and discrimination accuracy is mixed. Indeed, several studies have reported that fast-style individuals do not make more discrimination errors than slower individuals (Amy et al. 2012; Raine and Chittka 2012; Lucon-Xiccato and Bisazza 2016; Chung et al. 2017; Kareklas et al. 2017; Greis et al. 2022).

In some cases, a lack of evidence for the cognitive styles hypothesis in studies that explore decision (or discrimination) success may be due to artifacts or limitations of experimental design, an aspect of this topic that has been raised by several authors (Healy et al. 2009; Kolm 2014; Rowe and Healy 2014; Thornton et al. 2014; Boogert et al. 2018). Estimating individual differences in behavior is certainly challenging, as individuals need to be tested repeatedly to obtain robust values of average behavior, and small differences in design can impact observed variation and final results (Biro et al. 2010; Näslund et al. 2015; Näslund 2021).

A previous study testing the cognitive styles hypothesis in threespine sticklebacks (*Gasterosteus aculeatus*) found that while some (bolder) individuals were consistently faster to reach an end-chamber in a maze, there was no significant cost to their decision success (also referred to as “decision accuracy”)—evaluated as whether the fish entered the rewarded chamber first (Mamuneas et al. 2015). However, as noted by the authors, their results may have been impacted by the design of their T-maze. Specifically, the lack of differences in success rates across individuals with different behavioral types may have been limited by the relative simplicity of the maze and its small size. The maze had 2 choice options (end chambers) and the length of arms in the maze was relatively short which may have influenced the ability to detect differences between individuals—especially as the performance of sticklebacks tested across 3 maze designs found that arm length in mazes may impact behavioral and cognitive performance (Jones et al. 2023).

We conducted this study to re-investigate the cognitive styles hypothesis in threespine sticklebacks, using a study design and procedure based on Mamuneas et al. (2015), but with larger maze dimensions (longer arms) and an additional arm (forming a plus-maze instead of a T-maze) to increase the complexity of the task. Our aim was to test the second prediction of the hypothesis which proposes that decision success varies with behavioral type, mediated by underlying speed-accuracy trade-offs. Specifically, we tested whether cognitive performance (decision success) would relate to individual differences in behavior across 2 measures of behavior: latency to emerge from a start chamber in the maze trials and activity in a separate open field assay. As per the cognitive styles hypothesis, we predicted that (1) individual latency to emerge from the start box (a measure of behavioral type) would show a positive relationship with decision success, as slow-style individuals are expected to be more accurate. Similarly, we predicted that (2) individual activity in the open field test, another common measure of behavioral type across individuals, would have a negative relationship with success in the maze, as more active individuals are expected to exhibit more decision errors.

## Methods

### Subjects and husbandry

We collected 44 threespine sticklebacks (*Gasterosteus aculeatus)* from the Kinnessburn stream in St Andrews, UK using passive funnel traps set overnight for ~16 h as per Kressler et al. (2021). We selected fish based on size, ensuring all fish had body lengths between 4.5 and 5.0 cm (total length) and were not showing any breeding coloration. As fish were not in breeding condition, they were not sexed. Fish were collected and tested in 2 successive batches; as we only caught 19 appropriately sized fish in the first sampling event (Batch A), we then collected a further 25 fish (Batch B). Each fish was housed individually in a 45-L aquarium for 3 wk to allow acclimatization to the laboratory conditions before testing began. Each housing aquarium was aerated with an air stone and furnished with the same physical enrichment: gravel (mixed brown color, grain size 3–5 mm, 0.5 cm depth covering 100% of the bottom of the tank), one artificial plant (8 cm tall, light green leaves, 2 cm maximum leaf breadth), and an overhead shelter, in the form of a black opaque plastic sheet covering one-third of the surface area of the tank as shown to be preferred by sticklebacks (Jones et al. 2019). Housing and experimental water were maintained at 10.0 °C, with a 12L:12D photoperiod.

Fish were fed daily with frozen bloodworms at the end of the day, after any trials were completed. After testing, fish from batch A were held in a large housing tank until Batch B fish had been caught and tested—after which both batches were released at the location of capture.

### Setup—maze assay

**Fig. 1.**
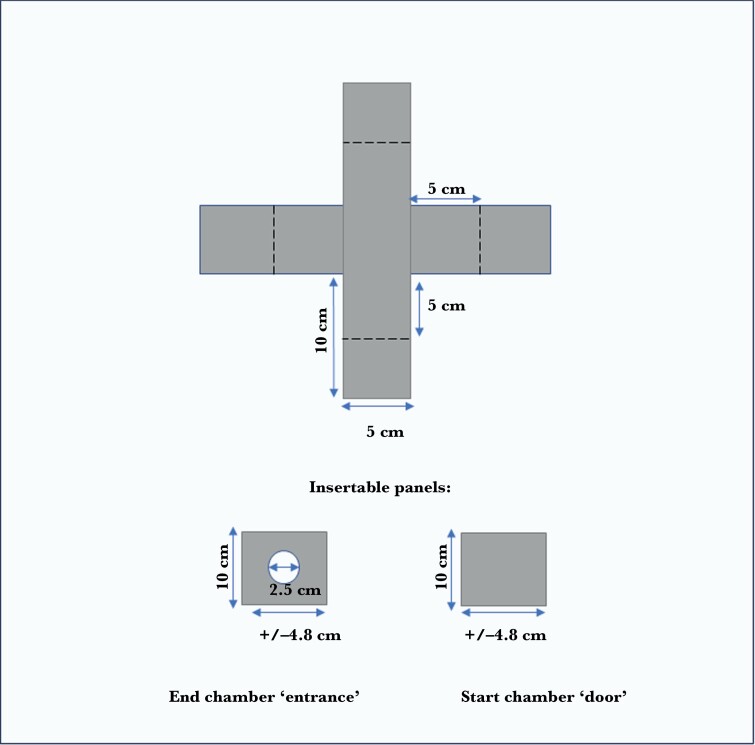
**A diagram of the dimensions of the mazes used in the study**. Dotted lines represent where insertable panels were positioned. Fish were placed in a start chamber with “door” panel inserted. At the start of the trial, this start chamber panel was removed to allow the fish to swim freely within the maze.

We used 4 identical plus-mazes to run 4 trials simultaneously. The mazes had opaque gray plastic walls and their physical dimensions were based on the previously used T-maze (Mamuneas et al. 2015) but modified (Fig. 1). The lengths of the arms were doubled from 5 cm to 10 cm and an additional arm was added resulting in a plus-shaped maze which has been validated in a study that compared behavioral and cognitive performance across maze designs (Jones et al. 2023). The maze walls were 10 cm high, and during trials, the water depth was kept at 8.5 cm. Each arm in the maze ended in a small chamber with a removable panel insert. The starting chamber in the central arm of each maze had an insert such that fish could not view or explore the maze until the insert had been removed. The remaining 3 inserts had a circular “entrance” cut into the middle (2.5 cm diameter) that allowed the fish to swim through, while preventing them from seeing the contents of the chambers prior to entering as per Mamuneas et al. (2015).

### Procedure—maze assay

All 44 fish (both Batch A and B) were tested in the maze assay in 25 repeat trials per fish. Before each trial, a single bloodworm reward was placed in one of the end chambers. The location of the reward was consistent for each fish across trials, and fish were randomly assigned to either “left rewarded” or “right rewarded” before trials started; the center “top” arm was never used as a rewarded position as using it as a reward position can introduce bias (Jones et al. 2023). Trials were run 4 at a time under a single overhead camera (USB 5-megapixel). Each fish was first placed in the “start chamber” of their respective maze and left for 30 seconds. The recording was then started, and the start chamber “door” partition was removed and placed over the start chamber to provide a shelter, allowing the fish to explore the maze to locate the food reward. After 12 min, the trial was ended, and the fish were returned to their housing tanks. The mazes were rinsed out and refilled with new aerated water from a reservoir tank within the fish room to mitigate potential scent biases affecting consecutively tested fish. Each fish was tested once per day for 5 wk, with a 2-d break over each weekend, resulting in a total of 25 trials per fish. This 2-d break from trials over each weekend was conducted as per Mamuneas et al. (2015).

### Setup—open field test

**Fig. 2.**
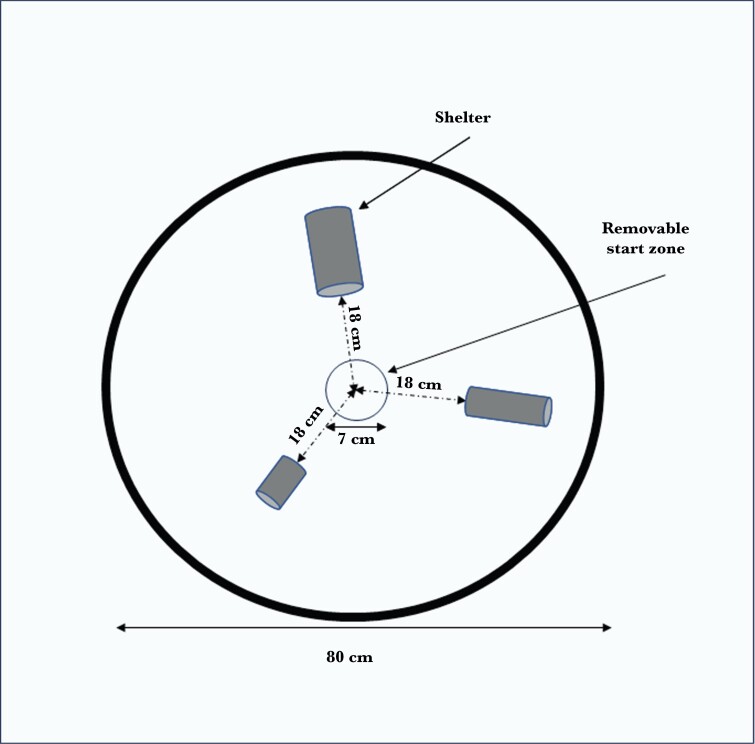
**Design and dimensions of the open field arena**. The circle in the center represents the transparent cylindrical holding chamber where fish were introduced prior to starting the trials. This holding chamber was removed to start the trial and allow the fish to swim freely within the arena.

We used 2 arenas to run 2 open field assays simultaneously. The setup was used in a previous study (Jones et al. 2024). These were 80 cm diameter circular pools (plastic paddling pool) with 20 cm high, opaque black walls (Fig. 2). The bottom was covered in fine white sand (grain size: 1 to 2 mm) for ease of visually tracking the fish, and water depth was kept at 10 cm from the top of the sand. Three PVC tubes of different sizes were provided for shelter. A wide-angle video camera (USB 5-megapixel) was mounted directly above each arena to record a top-down view of the fish behavior.

### Procedure—open field test

We tested each of the 25 individuals from the second batch (B) of fish in the open field test (OFT). We did not use Batch A, as we ran this test after the maze trials, when these individuals had already been moved to group housing due to tank availability limitations, and identifying individuals was not possible. Each fish was tested once a day for 4 d. For each trial, a single fish was placed in the midpoint of the arena in a circular transparent plastic chamber (7 cm in diameter) prior to starting the trial. The fish were held there for ~30 s before the chamber was removed to start the trial. Trials were recorded using a video camera (USB 5-megapixel) for a 10-min period. Fish were then transferred back to their respective housing tanks. The position of the shelters within the arena was altered between trials to control potential side biases; however, they were always equidistant (18 cm) from the center of the arena. The water was drained and refilled between trials, with new aerated water from a reservoir tank kept at the room temperature (10.0 °C) to mitigate scent biases affecting consecutively tested fish. After water refills, the sediment was smoothed and flattened to a uniform depth by hand (with latex gloves).

### Measurements

We were interested in measures of cognitive performance, and separately, measures that could be used to estimate behavioral types of individuals (Table 1). To reduce potential bias, the maze trial videos were scored manually by a researcher who had no knowledge of the open-field test results, and the open field test was scored by another researcher, who had no knowledge of the maze results.

**Table 1.** The measures of behavioral type (both proxies sometimes used for boldness) and cognitive performance used in this study.

Term	Source of measure	Details of measure
Behavioral measures		
Latency to emerge from the start chamber	Maze trials	Time, in seconds, from trial start (chamber door panel raised) until the fish left the start chamber.
Time spent actively swimming in the open	Open field trials	Time, in seconds, spent actively swimming (moving 1 body length per second) more than 1 body length away from the arena walls.
Cognitive performance		
Decision success	Maze trials	Binary, whether the first end chamber entered in a trial was the “correct” rewarded one, or not.

For the maze trials, we used video recordings to score 2 measures for each trial: (1) latency to emerge from the start chamber (latency) was recorded as the number of seconds taken until the fish’s whole body had emerged, which is commonly used as a measure of behavioral type—specifically boldness (Burns 2008; Webster et al. 2008; Alfonso et al. 2019; Näslund 2021) and (2) decision success (binomial) was recorded as whether the fish entered the rewarded chamber first or not. On some occasions, fish left the start chamber within the first second in a “flight” response and then froze in position, which is a relatively common response to being startled (Näslund et al. 2015). In these instances, we scored latency as time to begin swimming freely (this occurred in 42, or 0.04% of all trials). Trials where fish did not enter any chamber were excluded from analysis, which amounted to 89 trials (8%), the majority of which were in the first 4 d of tests.

For the arena test, we scored all videos using the BORIS software (Friard and Gamba 2016). We measured the duration of time spent actively swimming (moving at least 1 body length per second) more than 1 body length away from the arena walls over the course of the 10-min trials.

### Analysis

All analyses were conducted using R version 4.1.3 (R Core Team 2022). The lme4 package (Bates et al. 2015) was used to fit the univariate generalized linear models. To calculate estimates of repeatability, “R,” we used the “rptR” package (Stoffel et al. 2017). Multivariate mixed-effects models were fitted using the R package MCMCglmm (Hadfield 2010).

We first obtained repeatability estimates for the 2 behaviors of interest to provide evidence for consistent among-individual differences. We ran Rptr analysis on the latency to leave variable across all maze trials, and the time swimming in the open variable from the open field tests. In both instances, we used 1000 bootstraps to calculate repeatability across time (trials) for each behavioral trait measure.

To address our primary question about the relationship between behavioral type and cognitive performance, we fitted a Bayesian bivariate mixed effect model, following the approach recommended for capturing among-individual covariation by Houslay and Wilson (2017). Here, latency to emerge from the chamber was fitted with a Gaussian error, and decision success was fitted using the “categorical” error family. For both traits, we incorporated fixed effects of the batch, week, day of the week, and the interaction of week and day of the week. Note, that given the expected differential learning rates and thus changing success over time, we considered including the trial number in the model. However, to account for the potential impacts of the 2-d break over the weekend when no trials were run, we used the “Week” and “Day of Week” terms instead to capture change in success over time. Including “Day of the week” (and its interaction with “Week”) allowed us to capture potential within-week changes in motivation across the 5 d of testing (and consecutive food rewards), and changes after the 2 d without trials and associated food rewards. We retained all terms in this model as we are most interested in assessing the among-individual variances and covariances. In the random effects, we allowed both the intercept and “Week” slopes to vary among individuals for both traits and covary between traits (i.e. estimating a 4 × 4 covariance matrix at the among-individual level). Behavioral measures were allowed to covary at the residual level, although the residual variance for decision success was fixed at a value of 1 due to the use of the categorical error family. We used relatively uninformative priors on the variances and covariances, with parameter expansion for the random effect terms.

Note that the choice of intercept positioning (i.e. where x = 0) is of high importance in our study. This is because the use of interactions in fixed effects and slope variability in the random effects means that intercept positioning may have a strong influence on the estimation (and subsequent interpretation) of variances and covariances. The batch effect is centered between the 2 batches, and the day of the week is centered at the midpoint of the 5 d. For latency, we center the week effect at the midpoint of the 5 wk to assess average latency. However, for decision success, we set the final week as 0 such that intercept and slope (co)variances are estimated at the end of the testing period.

To investigate whether activity in an open field test was related to decision success in the maze we included “time in the open” as a third response variable in a multivariate extension of the above model. For this trait, we fitted a fixed effect of trial number (centered at the middle trial) and allowed random intercepts for variation among individuals (and covariation with intercepts and slopes for other traits as described above). For both multivariate models, diagnostic plots and multiple runs were used to assess model fit and convergence, a number of iterations were set to 220,000, the burn in was set at 20,000, and thinning interval at 50. We report variances, covariances, and correlations as the median and 95% highest posterior density interval from the posterior distributions.

### Ethical note

The experiments adhered to the “Guidelines for the treatment of animals in behavioral research and teaching (ASAB 2012).” The procedures performed were in accordance with the ethical standards and the project was approved by the University of St Andrews Ethics Committee. The principal source of potential stress was in transferring fish between tanks and the testing arena. Only experienced handlers caught the fish, and transfer time was kept to a minimum. Fish were monitored for at least 15 min immediately after any movement between tanks. All fish maintained a healthy appetite throughout the study. All individuals were retained in the laboratory for a further 3 wk, and as none showed visible symptoms of disease they were returned to the point of capture.

## Results

### Inter-individual repeatability

Both of our behavioral measures of interest were repeatable at the among-individual level: repeatability estimates (R) were significant (*P* < 0.05) for both “latency to emerge” in the maze trials and “time spent actively swimming” in the open field trials (Table 2). However, variation in activity that can be attributed to among-individual differences in the open field test is markedly lower than that from the latency to emerge.

**Table 2.** Summary of results for the repeatability “R” analysis of behavior across trials in both experiments simulated from 1000 bootstraps.

Experiment	Variable details	R	SE	Lower CI	Upper CI	*P*-value (LRT)
Maze trials	latency to emerge from start chamber across 25 trials, *n* = 44 fish	0.495	0.058	0.362	0.593	<0.001
Open field test	time spent swimming in the open across 4 trials, *n* = 25	0.268	0.112	0.023	0.481	= 0.006

CI: confidence interval.

### Cognitive styles

Both decision success and latency to leave the start chamber showed evidence of among-individual variation in both intercepts and week-related slopes (Table 3). We found a strong negative correlation between intercepts and slopes for latency, such that those individuals with higher average latencies (taken from the midpoint of the trial period) tended to have a more negative slope, i.e. those that, on average, left the start chamber faster, more rapidly decreased their time to leave across weeks. The 95% credible interval does not include zero, indicating confidence in this negative relationship. We found a strong positive correlation between intercepts and slopes for decision success, such that those individuals with higher average success at the end of the trial period tended to have a more positive slope, i.e. those more successful at the end of the trial period showed greater improvement across weeks. The 95% credible interval does not include zero, indicating confidence in this positive relationship.

**Table 3.** Between individual variance–covariance–correlation matrix of random effects from the bivariate model of decision success and latency to emerge in the maze trials. Variances are shown in bold on the diagonal, with covariances above and correlations below (presented as medians and 95% credible intervals from the posterior distributions).

	Latency intercept	Latency slope	Success intercept	Success slope
Latency intercept	**0.63 (0.37,0.92)**	−0.11 (−0.19, −0.05)	0.63 (2.58, 1.32)	0.14 (−0.04, 0.35)
Latency slope	−0.66 (−0.84, −0.43)	**0.05 (0.03, 0.08)**	−0.06 (−0.24, 0.11)	−0.02 (−0.07, 0.03)
Success intercept	0.41 (0.06, 0.69)	−0.14 (−0.49, 0.21)	**4.09 (1.61, 7.11)**	0.79 (0.20, 1.61)
Success slope	0.37 (−0.08, 0.72)	−0.17 (−0.56, 0.26)	0.79 (0.54, 0.96)	**0.25 (0.07, 0.50)**

**Fig. 3.**
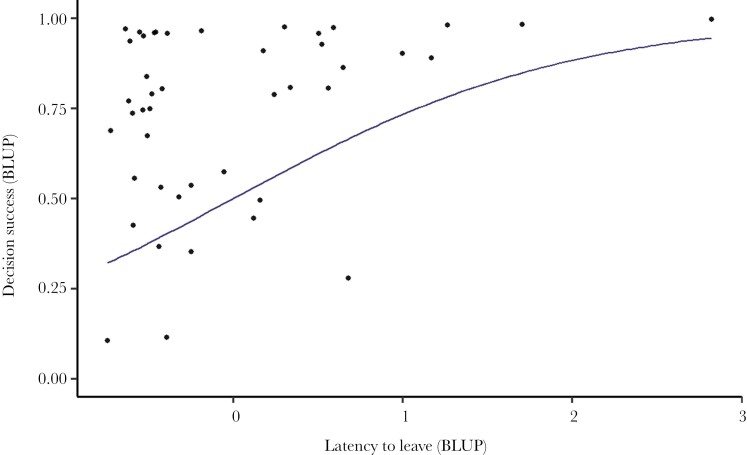
**The relationship between latency to emerge from the start chamber varies with decision success in maze trials.** The line shows the estimated regression slope (calculated as the among-individual covariance between these traits divided by the among-individual variance in latency, with predictions for this range of values transformed into probability space on the y-axis). Points show the best linear unbiased predictors (BLUPs) for individual fish latencies as estimated from the fitted multivariate model. For decision success, these were added to the overall intercept estimate from the model for transformation into probabilities. *N* = 44 fish, estimates from 25 trials.

We found a strong positive correlation between intercepts for latency and decision success, indicating that individuals who tended to have higher latency values also tended to have higher decision success (Table 3; Fig. 3). The 95% credible interval does not include zero, indicating strong confidence in this positive relationship.

**Fig. 4.**
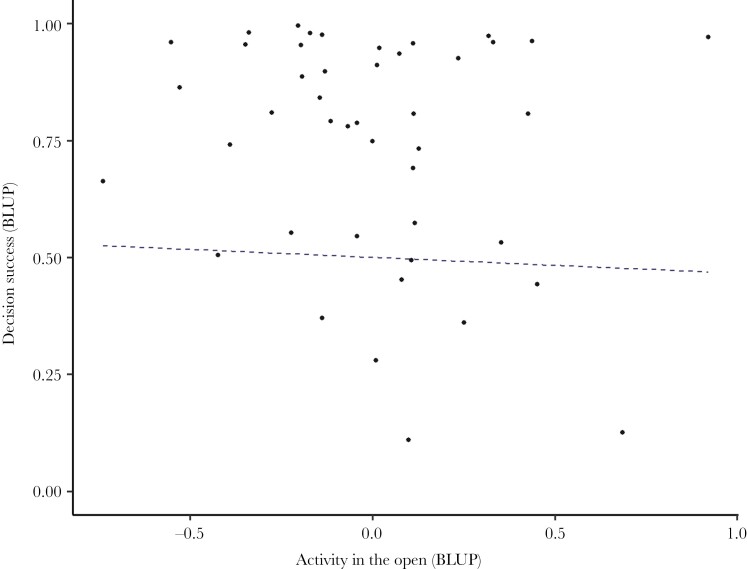
**No relationship between time spent actively swimming in the open field test and decision success in maze trials.** The dashed line shows the non-significant estimated regression slope (calculated as the among-individual covariance between these traits divided by the among-individual variance in time spent active in the open, with predictions for this range of values transformed into probability space on the y-axis). Points show the best linear unbiased predictors (BLUPs) for individuals as estimated from the fitted multivariate model. For decision success, these were added to the overall intercept estimate from the model for transformation into probabilities. Estimates from *N* = 25 fish across 25 maze trials and 4 OFT trials each.

We did not find evidence of a relationship between decision success and time spent actively swimming in the open (from the separate open field tests). While there was a weak negative relationship between the intercepts of decision success in the maze and time spent actively swimming, the 95% credible interval includes zero (β = −0.05, CI = −8.34 to 0.75; Fig. 4). Similarly, there was also no evidence for a relationship between the 2 measures of behavior: there was only a weak positive correlation between time spent actively swimming and latency to emerge from the start chamber in the maze trial, with the 95% credible interval including zero (β = −0.07, CI = −4.26 to 0.29).

## Discussion

We tested cognitive performance in threespine sticklebacks and found that decision success (frequency of correct first choices) was significantly related to one measure of behavioral type. In our study, individuals who took longer to leave the start chamber (sometimes termed less “bold” behavioral types) tended to be more successful in entering the rewarded chamber. These results suggest that variation in behavior at the level of the individual is associated with the cognitive performance of threespine sticklebacks, as predicted by the cognitive styles hypothesis (Sih and Del Giudice 2012). Our study joins evidence from an increasing number of studies showing that, in tests of spatial and other forms of cognition, there are trade-offs associated with an individual’s behavioral type and cognitive performance (Cussen and Mench 2014; Lucon-Xiccato and Bisazza 2017; White et al. 2017; Lucon-Xiccato et al. 2019; Jones et al. 2021). For example, great tits (*Parus major*) that made slower decisions were more accurate (Moiron et al. 2016), and in zebrafish (*Danio rerio*), slower fish were more accurate than faster individuals in a visual discrimination assay (Wang et al. 2015).

One potential reason for the disparity between our study and the previous study exploring the same question in this species is the maze design. Methodological design can influence our ability to detect meaningful inter-individual variation in cognitive performance. An important design choice is the number of cues or options available in a test; Chittka et al. (2009) theorized that presenting only 2 choice options in discrimination tests would be less challenging and result in a reduced ability to detect inter-individual differences in performance. The physical design of the setups used in studies may influence measures of cognitive performance in fishes as highlighted by several reviews (Hodges 1996; Benvenutti et al. 2021; Munson and DePasquale 2024). Certainly, the number of choices or options (and associated challenge complexity) has been shown to be important in studies exploring inter-individual variation in cognition as per the 2 preceding examples with great tits and zebrafish. Other studies that provide empirical support for cognitive styles typically use assays where subjects must choose between 3 or more options (Mazza et al. 2018; Jones et al. 2020; Rowell and Rymer 2021; Albers and Reichert 2022; Finkemeier et al. 2022). For example, a recent study showed that individual fish (minnows; *Phoxinus phoxinus*) with low metabolic rates, typically related to “slow” behavior types, showed better performance in more challenging f4-door mazes than fish with higher metabolic rates, while performing worse in less complex 2-door tests (Cortese et al. 2024). Similarly, male guppies, (*Poecilia reticulata),* tested in 2 escape-maze designs that differed in complexity (through differences in configuration) revealed evidence of learning only in the less complex maze (Prentice et al. 2022).

While differences in maze design might explain the differences in findings between this study and the previous study in sticklebacks which suggested faster individuals did not make more errors (Mamuneas et al. 2015), we did not explicitly test cognitive styles across different maze designs. We cannot, therefore, rule out other factors that may have contributed to the difference in results. For example, population differences may contribute to the disparity in findings between our study and the earlier one, as populations of the same species can show variation in behavior and related cognitive performance (Webster and Rutz 2020). The environment, both social and physical, can impact cognitive performance and even brain development in fishes. Studies have shown that the level of habitat complexity (Salvanes et al. 2013; Roy and Bhat 2016; Fong et al. 2019), and population density (Triki et al. 2019) can affect brain development and cognitive performance.

There are many causes of consistent behavioral differences between individuals and expressions of behavioral type that may have contributed to the cognitive styles we observed (Dall et al. 2004, 2012; David and Dall 2016). A host of underlying physiological and endocrine mechanisms can mediate individual behavioral differences, aka “personality” (Killen et al. 2012; Mazza et al. 2019; Kraus et al. 2020), including inter-individual differences in metabolic state (Borsook et al. 1978; Jones et al. 2005; Careau et al. 2008; Sih et al. 2015; Näslund 2021) and stress responses (Mesquita et al. 2015; Bensky et al. 2017; Alfonso et al. 2019; Mazza et al. 2019; Houslay et al. 2022). Sex is also an important factor. Many species can show marked sex differences in cognitive performance (Schuett and Dall 2009; Carazo et al. 2014; Lucon-Xiccato and Bisazza 2016; Szabo et al. 2019; Fuss 2021). For example, male guppies perform significantly worse than females in discrimination and reversal learning tasks (Fuss and Witte 2019). Similarly, Wallace et al. (2020), showed that male and female mosquitofish (*Gambusia affinis*) differed in both behavioral traits and cognitive performance, at least in some learning tasks. While we did not record sex (as the fish were not displaying full sexual characteristics at the time of the study), it is possible that the fast-style fish with lower latency to emerge and higher activity in our study were males. We raise this point as behavioral type has been found to correlate with sex in threespine sticklebacks in other studies: male sticklebacks were found to spend consistently more time out of cover than females in open field tests (King et al. 2013), and Mamuneas et al. (2015) found that males were more likely to be faster to arrive at the correct choice than females. A recent study exploring performance in a detour assay found that male sticklebacks outperformed females, with the authors suggesting that this was due to the males being less neophobic than females (Keagy et al. 2019). The question of what drives the relationships between an individual’s behavior and performance in cognitive tasks remains open.

We did not find a relationship between activity in the open field test and maze decision success. This may reflect a true absence of a relationship or reflect a confound between the potential traits that may (also) be captured in the test. It is still an open question as to how closely related activity and emergence are in sticklebacks (Näslund 2021), and the 2 measures may reflect different aspects of overall behavioral phenotype and different relationships with cognitive performance. Indeed, behavioral measures from open field tests can be interpreted as either exploration, stress, or activity, as highlighted by early tests with rats (Denenberg 1969). More recent work has shown that measures of traits such as “boldness” can be conflated across different tests (Burns 2008; Carter et al. 2012; David and Dall 2016; Lermite et al. 2017; Zidar et al. 2017). We would argue, however, that our results—and lack of relationship between activity score and cognitive performance—are more likely due to the limited number of repeat trials per fish (and limited sample of fish) that we were able to conduct in this assay. Studies have shown that robust estimates that more precisely reflect an individual’s behavioral type and take into account within-individual variation require greater length and number of trials (Biro 2012; Beckmann and Biro 2013). In our study, repeatability estimates at the individual level from the open-field assay were much lower than those in the maze trials.

In conclusion, we show that cognitive performance in threespine sticklebacks varies with inter-individual differences in behavior. We found that between-individual variation in decision success is associated with time to emerge from the start chamber, providing additional support for the cognitive styles hypothesis. Our study also reinforces the difficulty inherent to exploring questions in this area, with different measures of behavior in conjunction with testing methodologies showing conflicting results. Our study joins other empirical work highlighting the potential importance of the physical design of assays used in behavior and cognition (Swaney et al. 2024) and adds support to Chittka et al. (2009) suggestion that cognitive tasks with more than 2 choices (or cues) provide valuable insights in experiments that aim to explore cognitive variation between individuals.

## Data Availability

Analyses reported in this article can be reproduced using the data provided by Jones (2024). A publicly available copy of the data used in this study is also available at OSF at https://osf.io/ch7jr/?view_only = 35969f54391b4c2e9c55784936df05da
